# Huntington’s Disease: Complex Pathogenesis and Therapeutic Strategies

**DOI:** 10.3390/ijms25073845

**Published:** 2024-03-29

**Authors:** Huichun Tong, Tianqi Yang, Shuying Xu, Xinhui Li, Li Liu, Gongke Zhou, Sitong Yang, Shurui Yin, Xiao-Jiang Li, Shihua Li

**Affiliations:** Guangdong Key Laboratory of Non-Human Primate Research, Key Laboratory of CNS Regeneration (Ministry of Education), Guangdong-Hongkong-Macau Institute of CNS Regeneration, Jinan University, Guangzhou 510632, China; tonghuichun@stu2021.jnu.edu.cn (H.T.); yangtianqi12138@stu2021.jnu.edu.cn (T.Y.); xushuying@stu.jnu.edu.cn (S.X.); lxh1996@stu2022.jnu.edu.cn (X.L.); liuli66@stu2021.jnu.edu.cn (L.L.); zgk00@stu2022.jnu.edu.cn (G.Z.); yangsitong@stu2023.jnu.edu.cn (S.Y.); yinshurui20040416@stu2022.jnu.edu.cn (S.Y.)

**Keywords:** Huntington’s disease, huntingtin, aggregates, therapy, polyglutamine

## Abstract

Huntington’s disease (HD) arises from the abnormal expansion of CAG repeats in the huntingtin gene (*HTT*), resulting in the production of the mutant huntingtin protein (mHTT) with a polyglutamine stretch in its N-terminus. The pathogenic mechanisms underlying HD are complex and not yet fully elucidated. However, mHTT forms aggregates and accumulates abnormally in neuronal nuclei and processes, leading to disruptions in multiple cellular functions. Although there is currently no effective curative treatment for HD, significant progress has been made in developing various therapeutic strategies to treat HD. In addition to drugs targeting the neuronal toxicity of mHTT, gene therapy approaches that aim to reduce the expression of the mutant *HTT* gene hold great promise for effective HD therapy. This review provides an overview of current HD treatments, discusses different therapeutic strategies, and aims to facilitate future therapeutic advancements in the field.

## 1. Introduction

### 1.1. Huntington’s Disease

Huntington’s disease (HD) is an inherited neurodegenerative disorder caused by the abnormal expansion of CAG repeats in the huntingtin gene (*HTT*), which is located on chromosome 4p16 [1,2]. HD is characterized by a triad of motor, cognitive, and psychiatric symptoms. As the disease progresses, individuals may face challenges with speech, swallowing, and overall functional independence. The neuropathological hallmark of HD includes the selective vulnerability of medium spiny neurons (MSNs), which are GABAergic output neurons comprising over 90% of cells in the striatum [3], as well as the presence of intracellular aggregates of mutant huntingtin protein (mHTT). known as inclusion bodies.

HD is relatively rare, with an estimated prevalence of 5–10 cases per 100,000 individuals in most European countries, South America, North America, and Australia. However, the prevalence is significantly lower in Africa and Asia, with rates as low as 0.5 per 100,000 in Japan, China, and South Africa [4,5]. Although juvenile-onset HD cases can occur much earlier, the disease typically manifests in mid-adulthood, with an average age of onset between 35 and 50 years, significantly impacting patients’ personal and family lives. Patients often experience a life expectancy of 15–20 years following the onset of symptoms [6].

The underlying cause of HD is an abnormal expansion of CAG trinucleotide repeats in the *HTT* gene, where expanded CAGs code for polyglutamine stretch (polyQ). In normal individuals, there are typically 10–35 CAG repeats, while individuals with HD have more than 36 repeats. The length of the mutant polyQ expansion is inversely correlated with the age of onset of the disease. Adult-onset HD is associated with 40–50 repeats, while a juvenile form of the disease is associated with 50–120 repeats [7,8]. The expanded CAG repeats result in the production of mHTT with enlarged polyQ, which accumulates abnormally in neurons. The exact mechanisms by which mHTT leads to neurodegeneration are still uncertain, but it is believed to involve a combination of toxic gain-of-function effects and loss of normal HTT function [4]. The majority of evidence supports the hypothesis that polyQ-expanded HTT acquires a “toxic gain of function” [9]. Understanding the cause of HD and its disease pathogenesis will aid in the development of treatment strategies.

### 1.2. The Structure and Function of HTT

HTT is a large protein composed of 3144 amino acids and has an approximate weight of 348 kDa. Its evolutionary history can be traced back millions of years, with continuous expansion during vertebrate evolution. HTT exhibits a high degree of conservation among vertebrates, with the highest level of conservation observed [10,11,12,13]. It possesses several crucial functional domains, including the N-terminal polyQ region, the polyproline region, and three HEAT domains. Additionally, HTT contains nuclear export signals (NES) in the carboxy-terminus and nuclear localization signals (NLS). The NES in HTT is highly conserved across various species. However, N-terminal fragments of mHTT lacking NES or NLS have an increased tendency for nuclear localization by abnormally interacting with the nuclear pore protein translocated promoter region [14]. Recently, the structure of HTT in complex with HAP40 was successfully resolved using cryo-electron microscopy (cryo-EM) with a resolution of 4 Å [15]. However, studying unbound HTT presents challenges due to its large size, making it difficult to investigate using X-ray crystallography and cryo-EM techniques.

As a large protein, HTT has been found to interact with numerous proteins and participate in various cellular functions [16,17,18]. It is hypothesized to function as a scaffold in different cellular processes and plays a crucial role in the development of the central nervous system (CNS) [19,20,21,22]. Additionally, HTT has been reported to interact with transcription factors, coactivators, and transcriptional repressors, thereby regulating the transcriptional levels of genes and influencing various cellular physiological processes [23,24,25].

HTT also plays a vital role in intracellular transport through its interactions with molecular motor protein complexes. By interacting with various endocytic/trafficking proteins such as α-adaptin, HIP1, HIP14, HAP1, HAP40, PACSIN1, SH3GL3, clathrin, and dynamin, HTT is implicated in both long- and short-range axonal transport as well as vesicle trafficking processes [26,27,28]. Furthermore, HTT plays a crucial role in the formation and maintenance of synapses, influencing neurotransmitter release and synaptic transmission [29,30]. For instance, HTT has been found to interact with the SH3 domain of PSD95, a critical protein involved in synaptic transmission, suggesting its involvement in regulating synaptic plasticity [31].

Additionally, HTT has been reported to be associated with cell survival and apoptosis signaling pathways [32,33]. It exerts anti-apoptotic effects by binding to Pak2, inhibiting its cleavage by caspase-3 and caspase-8. This prevents the transformation of Pak2 into a mediator of cell death [34]. Knockdown of HTT in neuroepithelial cells of the neocortex disrupts cell migration, reduces proliferation, and increases cell death, particularly during early neural development [35].

HTT also interacts with proteins involved in cellular cytoskeleton and motility, playing a role in cytoskeleton assembly and the regulation of cell morphology. These functions contribute to cell movement and migration [36]. Additionally, HTT has been identified as a component of the pathway that regulates the orientation of mammary stem cell division. Depletion of HTT in basal progenitors leads to mitotic spindle misorientation [37]. In neurogenesis, HTT acts as a regulator that helps maintain the proliferative potential of precursor cells [38].

### 1.3. Pathogenic mHTT Forms

HTT contains a region in its N-terminal called PEST sequences, consisting of prolines (P), glutamic acid (E), serine (S), and threonine (T), which makes it susceptible to cleavage by various disease-related or pathologically upregulated proteases like calpain, caspases, and lysosomal proteases [39,40,41,42,43,44,45,46]. The N-terminal fragment or exon1 of mHTT containing polyQ repeats can be generated through proteolytic cleavage, and it is widely accepted that this cleavage process plays a significant role in the formation of mHTT N-terminal fragments and subsequent aggregates [47].

On the other hand, an excessive number of CAG repeats can affect the splicing of mutant *HTT* (*mHTT*), resulting in the generation of a truncated transcript known as HTT exon1 through abnormal splicing [48]. Aberrantly spliced HTT exon1 has been observed in both HD mouse models and human tissues, and a recent study using an HD knock-in (KI) pig model also detected it in the striatum, cortex, hippocampus, and cerebellum [49]. However, its expression level in the HD KI pig model was significantly lower than that of normally spliced *HTT* exon products. Thus, the contribution of mHTT N-terminal fragments or mHTT exon1 products produced by aberrant splicing to the pathogenesis of HD requires further investigation.

The abnormal N-terminal fragment and HTT exon1 protein can undergo misfolding and aggregation, leading to more severe cellular dysfunction and toxic effects [50,51,52]. Based on this evidence, some researchers propose a novel approach to reducing mHTT toxicity by modifying the proteolytic cleavage of HTT and decreasing the formation of more toxic N-terminal HTT fragments [53].

The aggregation of mHTT is a prominent pathological feature of HD. mHTT can spontaneously aggregate, forming various structures such as monomers, oligomers, and inclusion bodies composed of fibrils [54,55]. Initially, the N-terminus of mHTT undergoes conformational changes, adopting a misfolded structure rich in β-sheets, which promotes self-association and aggregation. During the aggregation process, small oligomeric intermediates are formed, as observed in human HD brains and HD mouse models [56]. These stable oligomers then act as seeds for the formation of mHTT fibrils and large inclusion bodies in the cytoplasm and nucleus [57]. The expanded polyQ region in HTT leads to an elongation of the random coil structure, further promoting protein aggregation [58]. The aggregates assemble into fibrils by recruiting new monomers, accelerating their growth. The elongating fibrils can induce nucleation-dependent fibrils, resulting in the formation of fibril branches [55]. These insoluble fibril-rich inclusion bodies accumulate within affected cells. Previous studies have shown that oligomers often selectively target proteins with extended, low-complexity sequences, potentially disrupting crucial cellular pathways. However, soluble forms of mHTT engage in more extensive and detrimental interactions compared to insoluble aggregates [59].

Large inclusion bodies containing mHTT were previously considered pathogenic [60,61]. However, it is interesting to note that large mHTT inclusion bodies can occur without causing cell death, and conversely, cell death can occur without the presence of inclusion bodies. This has led to the idea that mHTT oligomers are more toxic [62,63], while the subsequent formation of inclusion bodies may be protective [64,65,66]. Small mHTT oligomers and fibrils have been observed in the brains of HD patients, serving as precursors to larger inclusion bodies [63]. In mouse and fruit fly models of HD, the formation of mHTT oligomers and fibrils occurs before symptom onset and increases with disease progression. Notably, many neurons undergo cell death without forming inclusion bodies, and the formation of inclusion bodies has been shown to enhance survival and decrease levels of mHTT in primary cultures of rat striatal neurons [67]. Therefore, the question of whether the formation of mHTT inclusion bodies represents a protective mechanism or enhances toxicity remains a subject of debate.

## 2. The Pathogenetic Mechanisms of HD

Proteins within cells do not function in isolation; instead, they organize into either stable or transient protein complexes to carry out their cellular functions [68,69]. Abnormal aggregation of HTT has a profound impact on protein–protein and protein–DNA interactions, as well as organelle functions within cells. This abnormal aggregation disrupts these interconnected processes, which mutually influence each other.

### 2.1. Abnormal Protein–Protein Interactions

Recent studies have revealed that both mutant and wild-type HTT (wtHTT) interact with a large number of proteins [70]. HTT interacts directly or indirectly with numerous intracellular proteins involved in protein translation, signal transduction, membrane trafficking, and chromatin organization. Many of these protein–protein interactions depend on the polyQ sequences present in HTT, such as interactions involving HAP1, HAP40, HIP-1, syntaxin-1B, vesicle-associated membrane protein 2, SNAP25, NSF, and synapsins 1 and 2 [71,72,73]. The stability and levels of these protein interactions are altered by mHTT, potentially leading to the dysregulation of intracellular signaling pathways, gene expression, synaptic function, and cellular functions [74]. Moreover, the abnormal protein–protein interactions induced by mHTT have been associated with alterations in brain cholesterol homeostasis [75,76,77,78]. mHTT has been found to bind to the SREBP2/importin β complex, preventing the nuclear import of SREBPs involved in cholesterol biosynthesis [79]. This sequestration of SREBPs in the cytoplasm hinders the upregulation of cholesterogenic genes under sterol-depleted conditions. Additionally, mHTT interacts with proteins involved in protein translation, such as Mapk3, Eif3h, and Eef1a2, potentially disrupting the protein translation process [80].

### 2.2. Transcriptional Dysregulation

The mechanisms underlying transcriptional dysregulation in HD are multifaceted. The toxic mHTT can impact the transcriptional process through various means, including disruption of normal protein–protein interactions with the transcriptional machinery and modification of chromatin structure or genomic DNA, resulting in aberrant gene expression [81]. The polyQ repeats present in mHTT have a propensity to form insoluble aggregates. These aggregates can interact with glutamine-rich activation domains found in various transcription factors, such as cAMP response element-binding protein (CREB), Sp1, and the transcriptional coactivator CREB binding protein (CBP) [82,83,84,85]. The abnormal interaction between mHTT and SP1, for example, downregulates the transcript level of the metabolic enzyme cystathionine γ-lyase in HD models. Furthermore, mHTT has been shown to abnormally interact with crucial components of the core transcriptional machinery, including the large subunit of RNA polymerase II and TATA-binding protein (TBP) [86,87]. The expansion of polyQ can enhance HTT-DNA interactions, particularly with recognition elements of transcription factors whose function is disrupted in HD, leading to abnormal expression of specific mRNA species.

### 2.3. Mitochondrial Dysfunction

Mitochondrial dysfunction has been identified as an early pathological mechanism underlying the selective neurodegeneration observed in HD [88]. The abnormal aggregation of HTT is associated with mitochondrial dysfunction. Studies have shown that mHTT disrupts mitochondrial function by suppressing the expression of PGC-1α, a transcriptional coactivator responsible for regulating various metabolic processes, including mitochondrial biogenesis and respiration [89]. This disruption can lead to increased production of reactive oxygen species, resulting in cellular damage and cell death [90].

### 2.4. Autophagy Dysfunction

Research has shown that aggregated mHTT can disrupt the normal functioning of the autophagy pathway, resulting in the accumulation of waste materials and impaired protein degradation [91]. A recent study comparing gene expression profiles between striatal neurons directly reprogrammed from fibroblasts of HD patients and healthy controls observed a significant down-regulation of genes enriched in the autophagy–lysosome pathway [92]. J3, identified as an autophagy inducer through high-content screening, has shown promising potential as an antibody therapy for HD. In vivo studies have demonstrated that J3 treatment leads to a reduction in the levels of mHTT in the striatum. Additionally, J3 treatment increased the levels of DARPP-32 [93]. Moreover, both in vitro and in vivo investigations have highlighted the potential of autophagy activators, such as rapamycin, lithium chloride, and trehalose, for the treatment of HD. These activators have shown promising results in promoting autophagy and reducing the accumulation of mHTT [94,95,96,97,98].

### 2.5. Proteasomal Dysfunction

The presence of ubiquitin in inclusion bodies, which are characteristic of HD, allows their detection using antibodies against ubiquitin and proteasome. Extensive evidence has shown that the ubiquitin–proteasome system (UPS) function is impaired in various HD models [99,100,101]. It has been proposed that the sequestration of proteasomes by mHTT aggregates contributes to the alterations in UPS activity by recruiting or sequestering key components of the UPS [102,103].

### 2.6. Excitotoxicity at Extrasynaptic NMDA Receptors

HD is associated with enhanced N-methyl-D-aspartate (NMDA)-induced excitotoxicity. In striatal tissue from young, excitotoxin-sensitive YAC mice and cultured MSNs, there is an amplified binding of GluN2B with PSD-95 [104]. This enhanced binding may be attributed to altered activation of the p38 MAPK/GluN2B/PSD-95 toxic signaling pathway [105]. Additionally, research has shown that increased synaptic STEP and calpain activation contribute to the disrupted localization of NMDAR in YAC128 mice [106].

### 2.7. DNA Damage Repair

The expansion of trinucleotide CAG/CTG repeats in somatic tissues is believed to contribute to the continuous progression of HD throughout the life of the affected individual [52,107,108]. Genome-wide screens conducted to identify disease modifiers have highlighted the significance of DNA repair genes, such as *FAN1*, *LIG1*, *MLH1*, *MSH3*, *PMS1*, and *PMS2*, in HD [109,110]. FAN1, a nuclease involved in DNA repair, has been associated with the delayed onset and slower progression of HD [111]. In addition, reciprocal congenic mice studies have pinpointed the *MSH3* gene as the key factor responsible for the differences in repeat instability [109]. A recent genome-wide association study on HD identified rs557874766, a single nucleotide polymorphism within a polymorphic 9 bp tandem repeat in the *MSH3*/*DHFR* region, as the variant with the most significant association with disease progression [112]. These findings provide support for the hypothesis that DNA repair pathways play a crucial role in mediating the impact of modifying genes on HD.

## 3. HD Treatment

Currently, HD does not have a cure, and the available treatments primarily aim to manage psychiatric and movement symptoms. Recent advances in understanding the molecular mechanisms of HD, coupled with the development of gene therapies and other emerging therapeutic approaches, provide hope for future treatments for this debilitating condition. We review the current state of potential therapies for HD, encompassing pharmacological interventions, strategies to reduce mHTT, stem cell transplantation, and gene therapy.

### 3.1. Pharmacological Interventions

Numerous pharmacological interventions have been developed to manage the symptoms of HD and improve the lives of affected individuals (Table 1). While current treatments provide symptomatic relief, ongoing research aims to develop more targeted and disease-modifying therapies to address the underlying cause of HD.

#### 3.1.1. Dopamine Modulation

Dopamine modulation plays a crucial role in the management of HD, particularly in addressing HD-associated chorea. The dysregulation of dopamine signaling in the basal ganglia, a brain region involved in motor control, forms the underlying principle of dopamine modulation in HD treatment [123]. In HD, there is a progressive loss of MSNs in the striatum, leading to an imbalance in the direct and indirect pathways of the basal ganglia circuitry. This imbalance results in dysregulation of dopamine release and aberrant signaling, contributing to the manifestation of motor symptoms, including chorea.

To address the dysregulation of dopamine modulation, dopamine transporter inhibitors have been approved for HD treatment. These medications selectively target and inhibit the DAT, which is responsible for the reuptake of dopamine from the synaptic cleft back into the presynaptic neuron. By inhibiting DAT, these medications reduce dopamine reuptake, leading to increased dopamine availability in the synaptic cleft. This increased dopamine concentration helps normalize dopamine signaling and alleviate motor symptoms associated with HD, such as chorea.

Tetrabenazine was the first dopamine transporter inhibitor approved for the treatment of HD-associated chorea. Subsequently, deutetrabenazine, a deuterated form of tetrabenazine, was developed to improve its pharmacokinetic profile. Both medications have demonstrated efficacy in reducing chorea severity and improving motor function in HD patients [124]. However, it is important to note that these medications have side effects, including akathisia, depression, and parkinsonism-like symptoms [125]. Therefore, careful monitoring and individualized dosing are necessary to optimize treatment.

#### 3.1.2. Glutamate Receptor Modulation

Glutamate receptor modulation has emerged as a promising therapeutic approach for treating HD [126,127]. Abnormalities in glutamate signaling, specifically excessive activation of NMDA receptors, have been implicated in the pathogenesis of HD, leading to excitotoxicity and neuronal damage. The principle behind glutamate receptor modulation in HD treatment lies in restoring the balance of glutamate neurotransmission and reducing excitotoxicity. Numerous compounds targeting glutamate receptors have been investigated in both preclinical and clinical studies.

One class of compounds includes NMDA receptor antagonists, such as memantine and amantadine. These medications act by blocking the excessive activation of NMDA receptors, thereby preventing excitotoxicity. Some studies have demonstrated that NMDA receptor antagonists can improve motor function and reduce neurodegeneration in HD [128]. However, the use of NMDA receptor antagonists to treat chorea remains highly controversial. A recent Enroll-HD study comparing trajectories in cognition over 5 years found no significant differences in cognitive performance between memantine users and non-users [129]. Thus, extended, larger-scale studies are needed to further validate the therapeutic potential of NMDA receptor antagonists.

Another approach involves modulating metabotropic glutamate receptors (mGluRs). mGluRs are G-protein-coupled receptors that regulate glutamate neurotransmission. Activation of certain mGluR subtypes, particularly mGluR5, has been implicated in HD pathology [130,131]. Animal studies have shown that mGluR5 antagonists can activate autophagy through convergent mechanisms, promoting the clearance of mHTT aggregates and reducing neurodegeneration in HD models [132]. Therefore, antagonists of mGluR5, such as AFQ056, have been investigated as potential therapeutic agents [133].

#### 3.1.3. Strategies to Reduce HTT Aggregation and Enhance mHTT Clearance

Modulating protein–protein interactions is a promising therapeutic approach for disrupting or modulating the interactions between mHTT and its binding partners, which play a crucial role in the pathogenesis of HD. This strategy aims to interfere with downstream signaling pathways and cellular processes, reducing the formation of toxic protein aggregates and mitigating their detrimental effects on cellular function. Various approaches have been explored to target protein–protein interactions in HD.

##### Modulating Protein–Protein Interactions

One approach involves the use of small molecules and peptides that can bind to specific regions of mHTT or its interacting partners, thereby disrupting their interactions [134]. These small molecules can be designed to target key domains or motifs involved in protein–protein interactions, such as the polyQ tract of mHTT. By blocking the interaction between mHTT and its binding partners, these molecules aim to prevent the formation of toxic aggregates and mitigate downstream cellular dysfunction. Small molecules, including high-affinity RNA aptamers, have shown promise in stabilizing the monomeric form of mHTT and preventing its aggregation [135]. These aptamers have also exhibited enhanced cell survival, highlighting their therapeutic potential.

In addition to small molecules, peptide inhibitors have been developed and tested in preclinical studies. Peptides offer advantages such as high specificity, potent biological activity, cost-effectiveness, and excellent membrane penetrability [136]. Bivalent HTT-binding peptides have been successful in disrupting the aggregation process of mHTT and preventing the degeneration of photoreceptor neurons in a Drosophila model [137]. Another peptide, the polyQ-binding peptide QBP1, identified by combinatorial peptide library screening, has been shown to effectively suppress polyQ-induced neurodegeneration and improve the phenotype in both the Drosophila model and R6/2 mice [138]. Additionally, a specific 23-amino acid peptide called P42, derived from HTT, has demonstrated the potential to inhibit the aberrant aggregation of mHTT and improve disease symptoms.

Another strategy to modulate protein–protein interactions is through gene targeting-based approaches, such as CRISPR-Cas9, antisense oligonucleotides (ASOs), RNA interference (RNAi), or gene overexpression, to specifically target and alter the expression of proteins involved in pathogenic protein–protein interactions. For example, exogenous expression of HTT interacting protein K (HYPK) has shown the ability to reduce aggregate formation and cytotoxicity caused by N-terminal mHTT in neuronal cell lines [139,140]. The protein complex formed by HTT and HAP1 plays a crucial role in regulating organelle transport along axons. The depletion of HAP1 through CRISPR-Cas9-mediated approaches has resulted in selective neuronal loss in the striatum of adult HD KI mouse brains [141]. Overexpression of HAP1 in MSNs has been shown to restore calcium entry, enhance EGFR signaling activity, and inhibit mHTT-mediated cytotoxicity [142,143].

##### Autophagy Activation

The autophagy–lysosomal pathway is the primary biological pathway responsible for clearing intracellular protein aggregates. Impaired autophagy, including defects in autophagosome trafficking [144] and autophagosome–lysosome fusion [145,146], has been implicated in the pathogenesis of HD [147,148]. Age-associated upregulation of miR-29b-3p has been shown to promote HD-MSN degeneration by impairing autophagic function through human-specific targeting of the STAT3 3′ untranslated region in reprogrammed MSNs from symptomatic HD patients [92]. Accumulating evidence suggests that enhancing the autophagy–lysosomal pathway can decrease mHTT levels and enhance cell survival in cellular and animal models of HD [149,150]. The principle behind autophagy activation in HD treatment is to enhance the clearance of mHTT aggregates, thereby reducing their toxic effects on neurons.

Several approaches have been designed to stimulate autophagy in HD. One approach involves the overall activation of autophagy pathways. For example, rapamycin and its analogs, known as mTOR inhibitors, have been shown to induce autophagy by inhibiting the mTOR signaling pathway [151]. In a fly model of HD, treatment with rapamycin has been found to reduce mHTT aggregates, protect against neurodegeneration, and improve motor function [152]. Meanwhile, the rapamycin analog CCI-779 has demonstrated the ability to enhance performance on various behavioral tasks and phenotypes including the rotarod test, grip strength test, wire maneuver test, and tremors. Additionally, it has been shown to reduce aggregate formation in HD-N171-N82Q mice [96]. The activation of AMPK can promote autophagy by phosphorylating and activating key autophagy-related proteins [153]. Compounds that activate AMPK, such as metformin, have shown promising results in preclinical studies, reducing mHTT aggregates and improving motor function in HD models [153,154,155]. In addition to small molecules, gene-based approaches have also been explored to enhance autophagy in HD. For instance, viral vector-mediated delivery of genes encoding autophagy-related proteins, such as TFEB, a well-known master regulator of autophagy and lysosomal biogenesis, has been shown to induce autophagy and reduce mHTT aggregates in animal models [156]. Furthermore, a decrease in the expression of Rhes, a striatum-enriched mTOR activator, was observed in the brains of both HD patients and HD mice. Interestingly, the administration of exogenous Rhes can improve motor deficits and ameliorate brain pathology in HD mice [157].

Another strategy is to target selective autophagy. With the assistance of a variety of receptors that can recognize their specific cargos and connect them to LC3 through their LC3-interacting regions, selective autophagy can selectively identify and target specific substrates, including mitochondria (mitophagy) and protein aggregates (aggrephagy). The initial discovery of p62/SQSTM1 as a selective autophagy receptor aiding in the clearance of mHTT aggregates has paved the way for further research in this area [158]. Subsequently, several additional receptors for aggrephagy have been identified, expanding our understanding of the selective autophagy process. Notable examples include optineurin (OPTN), Tollip, and TAX1BP1, which have been shown to mediate the degradation of mHTT aggregates [146,159]. Tollip overexpression has demonstrated protective effects against the toxicity of polyQ-expanded HTT in cellular models of HD [66]. In a recent study, Ma et al. discovered that the chaperonin TRiC subunit CCT2 acts as a novel receptor involved in regulating the selective autophagy of aggregation-prone proteins, including mHTT [160]. CCT2 exhibits specificity towards aggrephagy and promotes the autophagic degradation of solid protein aggregates.

Ongoing clinical trials are currently evaluating the safety and efficacy of autophagy modulators, including mTOR inhibitors and AMPK activators, in HD. It is crucial to consider that while autophagy activation holds promise, it may also come with potential side effects. Therefore, determining the optimal timing and duration of treatment is essential [161]. Moreover, researchers are actively exploring the development of selective autophagy modulators that specifically target mHTT aggregates while preserving normal cellular components, representing a promising avenue for future research in HD therapeutics.

##### Proteasome-Mediated Protein Degradation

Proteasome-mediated protein degradation is a therapeutic approach aimed at enhancing the clearance of mHTT by promoting its degradation through the UPS. The mechanism underlying proteasome-mediated protein degradation in the treatment of HD involves the recognition of ubiquitinated mHTT by the 26S proteasome, a large multi-subunit complex responsible for protein degradation [162]. The ubiquitin molecules attached to mHTT serve as a signal for proteasomal degradation. The pathogenic form of the mHTT protein is ubiquitinated via K48 in ubiquitin, which promotes HTT degradation. Inhibition of the proteasome has been shown to increase the number of HTT aggregates in induced pluripotent stem cells (iPSCs) derived from HD patients [107]. By enhancing the activity of the proteasome, the clearance of mHTT aggregates can be facilitated, reducing their accumulation and mitigating their toxic effects. Several E3 ubiquitin ligases, such as UBE3A/E6AP [163,164], CHIP [165], HRD1 [166], and the SCF complex [167], can directly polyubiquitinate HTT through K48-linked ubiquitin chains. This polyubiquitination enables the clearance of HTT by the UPS. The ubiquitination process mediated by K-48 linkage relies on Ube3a, whose expression decreases in the aging brain. However, overexpression of Ube3a in the brains of aged HD KI mice has effectively decreased the accumulation and aggregation of mHTT. Mutations in the parkin gene, another E3 ubiquitin ligase, are associated with familial Parkinson’s disease. Partial suppression of parkin exacerbates the clinical phenotype and the impact of mHTT in the striatal cells of R6/1 mice [168]. In addition to the three classes of enzymes involved in monoubiquitination, proteasome activators offer an alternative means to inhibit mHTT-induced neurodegeneration. PA28γ, a proteasome activator, has shown promising results when a virus overexpressing PA28γ is stereotaxically injected into the striatum. This approach leads to a significant increase in peptidyl-glutamyl preferring hydrolytic proteasome activity, reduced presence of ubiquitin-positive inclusion bodies in the striatum, and improved behavioral abnormalities in YAC128 HD mice [169]. Furthermore, other small compounds can activate the proteasome. For instance, researchers have identified a Food and Drug Administration-approved drug called desonide through small-molecule microarray-based screening. Desonide has demonstrated the ability to suppress mHTT toxicity in cellular and animal models of HD by destabilizing mHTT through enhanced polyubiquitination, specifically at the K6 site [170]. It is crucial to acknowledge that proteasome-mediated protein degradation is a complex process, and the long-term effects as well as potential off-target effects of modulating proteasome activity need to be thoroughly evaluated.

##### Intracellular Antibody (Intrabody)

Intrabodies are gene-based approaches engineered to interfere with mutant proteins by expressing recombinant antibodies, typically the antigen-binding fragment, within intracellular compartments such as the nucleus and cytoplasm. The first intrabody for HD became available in the early 2000s through the establishment of a large naive human spleen single-chain variable fragment (scFv) phage-display library [171]. Intrabodies exert their effects through several mechanisms [172]: (1) direct binding to the functional domain of the target protein to neutralize its activity; (2) disruption of the interaction between pathological proteins and crucial binding partners; and (3) redirection of the pathogenic protein to alternative subcellular compartments, such as the proteasome, for degradation.

Various protein domains of the soluble form of HTT/mHTT have been targeted by intrabodies, including the polyQ domain [173], polyP domain [174], N-terminal exon 1 domain [175], and mHTT aggregates themselves [176]. In a previous study, we engineered a specific HTT protein-targeting intrabody that can reduce the neurotoxicity caused by cytoplasmic mHTT and alleviate associated neurological symptoms. This intrabody inhibits the accumulation of mHTT in neuronal processes and facilitates its clearance in the cytoplasm [177]. Recently, we identified that the last 23 amino acids of the C-terminus of the heavy chain of our previous intrabody, named smaller intrabody 3 (SM3), can bind to mHTT. SM3, linked to the lamp1 signal peptide, selectively binds to mHTT and facilitates its delivery to the lysosome, promoting the degradation of soluble and insoluble forms of mHTT. Additionally, SM3 can alleviate abnormal behaviors in HD KI-140Q mice by enhancing lysosomal degradation activity. Intravenous administration of SM3 effectively reduces soluble and insoluble mHTT in the brains of HD KI-140Q mice, improving HD-related neuropathology and motor function deficits [178].

#### 3.1.4. Strategies to Target the Complement Pathway

The aberrant activation of the complement system and the resulting inflammatory responses are considered key factors in HD. A broad range of changes in complement components, at both the transcriptional and protein levels, have been identified in the brains of HD patients and various HD animal models. Notably, there is a significant increase in the mRNA levels of complement components such as C1q C chain, C3, C4, and C1r in the striatum of HD patients. This increase is accompanied by distinct immune reactivity for C1q, C3, and C4 in oligodendrocytes, astrocytes, and neurons [179]. Moreover, the use of 3-NP has been shown to result in a substantial rise in the levels of C3, C9, C5aR1, and C5aR2 in the striatum of rats [180]. These findings highlight the involvement of the complement system and inflammatory responses in the pathogenesis of HD, emphasizing their potential as targets for therapeutic interventions.

Recent studies have yielded significant insights into the pathophysiological mechanisms underlying HD, particularly underscoring the therapeutic promise of complement system modulation. Notably, interventions such as C1q blockade with antibodies or genetic deletion of complement receptors on microglia considerably alleviated cognitive deficits and early developmental impairments in visual discrimination learning observed in the zQ175 mouse model [181]. These measures not only enhanced excitatory impulses to the striatum but also significantly reduced synaptic damage. This progress marks a promising development in the therapeutic application of complement modulation. In the phase 2 clinical trial (NCT04514367), ANX005, a humanized monoclonal antibody developed to inhibit C1q, demonstrated clinical improvement in a subset of HD patients with high baseline C4a/C4 levels. This trial demonstrates the potential effectiveness of targeting the complement system, specifically inhibiting C1q, as a therapeutic strategy for HD.

#### 3.1.5. Strategies to Target Astrocytes

Astrocytes play a pivotal role in shaping the neuronal environment by providing essential nutritional and metabolic support, assisting in the removal of neuronal metabolites, and maintaining local ion balance [182]. Overexpression of Sphingosine kinase 1 in astrocytes has been shown to enhance autophagy and promote the clearance of mHTT in HD cell models [183]. In HD mouse models, targeted activation of specific astrocytic functions in the striatum through AAV virus injection has shown promising results. These included boosting UPS degradation and lysosomal function via JAK2-STAT3 pathway activation [184], or initiating the N-terminal active fragment of human SREBP2 to trigger cholesterol biosynthesis pathways [185], which have led to significant reductions in neuronal mHTT aggregates and improvements in behavioral phenotypes. The pathological mechanisms in HD also involve disrupted homeostasis and signaling processes mediated by the downregulated Kir4.1 receptor in astrocytes. Restoring impaired Kir4.1 receptors in R6/2 mice not only corrects abnormal calcium and glutamate signaling but also rescues affected MSNs, thereby improving motor deficits [186]. These findings underscore the potential of targeting astrocytic functions as a therapeutic strategy in HD, highlighting the importance of astrocytes in disease pathogenesis and treatment.

#### 3.1.6. Strategies to Stabilize Somatic Expansion

The expansion of the CAG repeat tract in somatic cells, a crucial contributor to HD, is thought to impact the disease’s progression. As a result, genes within the DNA mismatch repair (MMR) pathway, which influence the repeat expansion, may be considered potential targets for therapeutic interventions [187]. In a study involving 683 HD patients with extreme onset or phenotype relative to the length of the *HTT* CAG repeat, researchers performed exome sequencing and identified variants in FAN1 clustered in its DNA-binding and nuclease domains. Furthermore, FAN1 overexpression has been shown to reduce the expansion of CAG repeats in human cells and patient-derived neurons [188]. Prior research identified that the highly conserved SPYF motif at the N terminus of FAN1 can bind with MLH1. This interaction inhibits the assembly of a functional MMR complex, thereby contributing to the stabilization of CAG repeats [189]. These findings highlight the role of FAN1 in modifying the progression of HD by stabilizing the expanded HTT CAG repeat.

Among the components of MMR that have been identified as modifiers of HD onset, MSH3 has shown promise as a potentially safe and effective target for therapeutic intervention [109]. In a recent study, researchers discovered a fully chemically modified short interfering RNA (siRNA) that effectively suppresses the expression of *MSH3,* both in laboratory experiments and in living organisms. When the siRNA is synthesized in a divalent scaffold, it successfully inhibits the expansion of CAG repeats in the striatum of two mouse models of HD. Importantly, this silencing of *MSH3* does not have any adverse effects on tumor-associated microsatellite instability or the expression of other genes involved in the MMR pathway [190]. Previous research has demonstrated that the knockdown of endogenous TDP-43 in the striatum of HD KI mice resulted in an expansion of CAG repeats. This expansion was correlated with increased expression of MSH3 and MLH1. Additionally, inhibiting MSH3 and MLH1 effectively reduced this CAG repeat expansion [191]. These findings provide additional evidence supporting the role of DNA MMR mechanisms, specifically MSH3 and MLH1, in the somatic expansion of CAG repeats in HD.

The use of strategies to control instability in HD represents a potential approach to modify disease progression and offer long-term benefits, unlike symptomatic treatments. However, the concept of targeting instability as a treatment for HD remains a topic of debate. In a different study, potent divalent siRNAs (di-siRNAs) were employed to suppress MSH3 mRNA expression in HdhQ111 mice in a dose-responsive manner. The results revealed a direct relationship, with a proportionality constant close to 1, between the inhibition of somatic *HTT* CAG expansions and the in vivo expression of MSH3 protein, highlighting MSH3 as a critical factor in limiting the rate of somatic expansions. Interestingly, even with a substantial 75% decrease in MSH3 protein levels, there was no observable change in the accumulation of striatal nuclear HTT aggregates [192]. Thus, further research is necessary to definitively establish whether managing instability can positively impact HD treatment. The long-term consequences of stabilizing somatic expansion are not fully understood and could potentially result in damage to brain cells. Therefore, a comprehensive understanding of the implications of targeting instability in HD is crucial to assessing both the benefits and potential risks associated with this therapeutic approach.

#### 3.1.7. Other Therapies

Curcumin, a phytochemical commonly found in Asian food, has been shown to possess a wide range of beneficial properties, including antioxidant, anti-inflammatory, and anti-fibrogenic effects. Research has demonstrated that curcumin effectively alleviates disease symptoms in a Drosophila model of HD by suppressing cell death [193]. Inflammation is commonly observed in HD patients before the onset of symptoms. Certain drugs, such as neflamapimod [194] and minocycline [195], have been investigated for their potential to improve glial cell functions and prevent neuroinflammation in HD patients.

### 3.2. Cell Therapies

Stem cell therapies have shown promising advancements in the treatment of HD owing to the unique characteristics of stem cells, such as their capacity for self-renewal and differentiation into various cell types. Several types of stem cell therapies have been investigated in the context of HD. The most investigated stem cell therapies in HD include mesenchymal stem cells (MSCs), embryonic stem cells (ESCs), neural stem cells (NSCs), and iPSCs. Each type of stem cell therapy has its own advantages and challenges, and ongoing research aims to optimize their use for the treatment of HD.

#### 3.2.1. MSCs Therapy

One specific stem cell therapy is MSC therapy, which can be obtained from various resources such as the umbilical cord, amniotic fluid, bone marrow, and adipose tissue. MSCs were initially used therapeutically to treat severe graft versus host disease [196]. Adipose and bone marrow-derived MSCs have been widely studied and applied in stem cell transplantation therapy for HD [197]. These MSCs have the ability to release neurotrophic factors, which are molecules that support the growth, survival, and function of neurons. By secreting neurotrophic factors, MSCs can potentially promote neuroprotection, enhance neuronal survival, and support the repair of damaged neural tissue in conditions like HD. Several preclinical studies using rodent models have been conducted to investigate the effects of MSC transplantation. For example, MSCs isolated from the bone marrow of mice were transplanted intrastriatally into R6/2 mice, leading to significant behavioral and morphological improvements. These positive effects were likely attributed to an increase in BDNF [198]. Additionally, MSC treatment has shown potential in protecting against 3NP-induced enlargement of the lateral ventricles, decreasing oxidative stress, enhancing cell viability, and extending lifespan in an HD rat model [199]. Transplantation of normal human adipose-derived stem cells, another commonly utilized type of MSC, has also demonstrated the ability to reduce striatal atrophy in YAC128 mice [200].

Prior research has demonstrated that the use of cytokine-based neuronal inducers can enhance the differentiation of bone marrow MSCs into functional neurons, exhibiting spontaneous activity and maturing into an electrophysiologically active state [201]. Concurrently, another study using a stroke model established that human adipose-derived MSCs could differentiate into neuron-like cells, characterized by positive expression of MAP2 and Synapsin 1/2, and exhibiting electrophysiological activity, thereby aiding in the reconstruction of hippocampal neuronal circuits [202]. However, the existing body of research does not provide substantial evidence, and no studies reported have that MSCs can differentiate into neurons in HD. Consequently, the potential for MSCs to differentiate into mature neuronal or glial cells remains a subject of ongoing debate [203].

MSCs are generally considered to have low immunogenicity. Transplantation of both rat and human MSC grafts induced very few mast cells, T αβ-cells, and no T γδ-lymphocytes in rats [204]. The anti-inflammatory cytokines secreted by MSCs play a crucial role in increasing neuroprotection and reducing disease-induced neuroinflammation. These results indicate that transplanted MSCs are not expected to elicit a strong inflammatory response from the host. The ability of MSCs to modulate the immune response and promote an anti-inflammatory environment can be beneficial in neurodegenerative diseases accompanied by neuroinflammation.

#### 3.2.2. ESCs Therapy

ESCs have demonstrated the ability to differentiate not only into neurons but also into astrocytes, highlighting their diverse differentiation potential for enhancing neuronal function and promoting brain development. In a quinolinic acid-induced HD rat model, hESCs were differentiated into nestin-positive neural precursors and transplanted into the striatum. The transplanted hESC-derived neural precursors were detected in both the cortex and striatum, showing signs of neuronal differentiation. Moreover, the transplanted animals exhibited significant behavioral improvement [205].

Clinical trials have shown improvements in HD symptoms using ESCs. In one trial, human fetal neuroblasts were grafted into the striatum of five patients. PET scans revealed increased metabolic activity in various subnuclei of the striatum in three patients, indicating functional grafts. These patients experienced motor and cognitive improvements or maintained normal function, demonstrating functional benefits in their daily activities [206]. However, the condition of the remaining two patients declined similarly to the control group, indicating that the clinical application of ESCs is not always successful.

In a recent phase II randomized trial called the Multicentric Intracerebral Grafting in HD trial, the rate of motor score decline after transplantation was similar to that before transplantation. Additionally, 40% of the patients developed anti-human leukocyte antigen antibodies after transplantation. This trial did not demonstrate any clinical benefit, possibly due to graft rejection [207]. Another study involving the implantation of human fetal ganglionic eminence in HD patients showed no signs of tissue overgrowth at the implantation site [208]. Therefore, further research is needed to investigate the potential adverse effects of ESC transplantation, such as rejection and tumor formation.

Harvesting, transporting, storing, and preparing ESCs for transplantation present significant challenges. Additionally, the use of ESCs raises ethical concerns due to the destruction of human embryos during their derivation.

#### 3.2.3. NSCs Therapy

NSCs can differentiate into various types of neural cells, including neurons and glial cells [209]. In HD, specific types of neurons (such as MSNs in the striatum) undergo degeneration. NSCs transplantation can replace these lost neurons, restore the damaged neural networks, and have neuroprotective effects by secreting a variety of neurotrophic factors, such as BDNF. Additionally, NSCs can modulate the neural environment by reducing inflammation and promoting endogenous neurogenesis, which could be beneficial in the context of HD [210].

In previous research, NPCs derived from a specific human iPSC line, namely 1231A3-NPCs, were grafted into the striatum in a quinolinic acid (QA)-lesioned rat model of HD. The graft recipients demonstrated notable behavioral enhancements for a period extending up to 12 weeks. Furthermore, the transplanted 1231A3-NPCs not only substituted for some of the lost neurons but also boosted innate neurogenesis, mitigated inflammatory responses, and helped restore the impaired neuronal connections [210]. In a recent study involving the transplantation of human NSCs into HD zQ175 mice, significant behavioral improvements were observed, along with an increase in BDNF levels and a decrease in mHTT accumulation. Evidence from patch clamp recordings and single-nucleus RNA sequencing suggested that these human NSCs differentiated into diverse neuronal populations, including MSNs and interneurons. They also established connections, which contributed to enhancements in membrane and synaptic properties. These findings emphasize the therapeutic potential of human NSC transplantation for HD [211].

However, the process of deriving NSCs and differentiating them into the desired types of neurons is technically challenging and requires further refinement. Additionally, our current understanding of HD pathology is limited, and it is unclear whether replacing lost neurons can fully restore the complex neural networks that are disrupted in HD.

#### 3.2.4. iPSCs Therapy

iPSCs have emerged as another potential therapy for HD, complementing the use of embryonic and MSCs. Unlike MSCs, iPSCs hold significant promise for replacing degenerated neurons in affected brain regions, making them a more suitable option for transplantation.

Since their development in 2006, iPSCs have generated significant excitement due to their ability to model human diseases in cell models [212]. The concept behind iPSCs therapy is to generate healthy neurons and replace dysfunctional or damaged neurons in the brain. These transplanted neurons have the potential to integrate into the existing neural circuitry, restore normal function, and halt disease progression.

In animal models of HD, significant changes were observed in 3-NP-treated rats, including decreased optical densitometric measures in the striatum and enlarged lateral ventricles. However, rats treated with 3-NP and receiving iPSC transplants did not exhibit these deficits. The transplanted iPSCs were found to survive and differentiate into region-specific neurons in the striatum, indicating a potential therapeutic effect [213]. However, the transplantation of iPSCs carries the risk of over-proliferation if the transplants are not pre-differentiated towards a specific germ layer, such as the neuroectoderm. To address this concern, researchers have shifted their focus to iPSC-derived NSCs, which are generated by differentiating iPSCs. This therapy has the potential to replace damaged brain tissue as it can differentiate into neurons [214]. In a prior study, iPSC-derived NSCs were transplanted into the striatum of YAC128 mice. The results of the study demonstrated not only the survival and differentiation of numerous iPSC-NSCs into striatum-specific MSNs but also the absence of tumor formation. These findings suggest that this approach could be a promising strategy for neuronal replacement therapy in HD [215].

iPSC-based therapy offers several advantages for the treatment of HD. One significant advantage is the patient-specific approach, where iPSCs can be derived from the patient’s cells. This personalized treatment approach minimizes the risk of immune rejection and enhances the compatibility of the transplanted cells. Additionally, pluripotent stem cells have the remarkable ability to self-renew indefinitely, providing an unlimited cell supply for transplantation and addressing the ethical concerns associated with embryonic stem cell use. Moreover, iPSCs can be utilized to create disease-specific cellular models, allowing researchers to study the mechanisms underlying HD [52,107,108]. While the results of iPSC-based therapy in preclinical and clinical trials have shown promise and demonstrated several advantages, it is important to acknowledge that iPSC therapies for HD still face challenges and require various improvements. Safety concerns are of utmost importance, as there is a risk of tumor formation. Additionally, generating a pure population of functional neurons from pluripotent stem cells is a complex task that requires careful optimization of differentiation protocols. By addressing safety concerns and optimizing differentiation efficiency, pluripotent stem cell-based therapies hold significant potential for the treatment of HD.

#### 3.2.5. Gene-Edited Autologous Transplantation

Autologous stem cell transplantation, which significantly reduces the risk of immune responses, is gaining increasing interest. As these cells, derived from patients, still carry the HD gene, it may be crucial to eliminate or silence the mutant HD gene. Numerous studies have successfully lowered mHTT levels [216], opening the possibility for gene-corrected autotransplants and thereby enhancing stem cell therapy’s effectiveness. One such investigation used allele-specific PAM-altering gRNAs in three NPCs from an iPSC line to remove the CAG expansion mutation. This led to complete mutant allele inactivation without affecting the normal allele, effectively halting mHTT production [217]. In a different study, the *HTT* gene was corrected in human iPSCs using a CRISPR-Cas9 and piggyBac transposon-based approach. The results showed that both HD and corrected isogenic human iPSCs could differentiate into active, excitable forebrain neurons, and the phenotypic defects observed in HD human iPSC-derived neural cells were successfully addressed [218]. While the idea of using autologous stem cells for HD treatment is promising, it’s worth noting that this field is still emerging, and further research is required to fully understand the safety and efficacy of this approach.

#### 3.2.6. “Prion-like” Propagation following Transplantation

During the process of transplantation, particularly in the context of neurological disorders, there are inherent risks, one of which includes the potential for prion-like transmission. In HD, this primarily involves cells or aggregates carrying the abnormal protein mHTT. These abnormal proteins may spread to transplanted tissues or cells, creating a situation similar to prion disease transmission. Some studies have demonstrated that mHTT aggregates could transfer to transplanted cell grafts in a prion-like fashion. For instance, human iPSC-derived MSNs were transplanted into the striatum of R6/2 mice, leading to a reduction in motor deficits and an increase in lifespan. However, mHTT aggregates were detected in the nuclei of transplanted cells [219]. Similarly, fetal striatal allografts transplanted into HD patients exhibited mHTT aggregates within the transplanted tissue [220]. These observations suggest the potential transfer of mHTT from the host to transplanted cells. Furthermore, research has shown that mHTT aggregates can migrate from the transplant to the host tissue. For instance, when HD skin fibroblasts were transplanted into healthy mice, mHTT aggregates were detected in host cells [221]. The spread of mHTT aggregates to healthy cells following transplantation in HD stem cell therapy could have adverse effects on the patient’s condition, potentially accelerating disease progression. Therefore, it is imperative for researchers to thoroughly evaluate and monitor this risk of transmission.

### 3.3. RNA Targeting Therapy

Given the complex pathological mechanisms of HD and the diverse effects of mHTT on a wide range of cellular functions, it is widely accepted that an effective therapeutic strategy would involve gene therapy to eliminate or reduce the expression of mHTT. Researchers have made significant progress in animal models and clinical trials, exploring various approaches for gene therapy in HD, including the following.

#### 3.3.1. SiRNA Treatment

Synthetic siRNAs can be specifically designed to target and degrade *HTT* mRNA, effectively reducing the production of mHTT protein. In a study, cholesterol-conjugated siRNA duplexes (cc-siRNA) were introduced into the striatum of mice carrying the *mHTT* gene, resulting in the prolonged survival of striatal neurons, reduced neuropil aggregates, decreased inclusion size, and improved performance on the balance beam test [222].

Silencing gene expression throughout the brain using siRNAs has been challenging, but a specific di-siRNA has shown promise in achieving potent and sustained gene silencing in the CNS of mice and nonhuman primates. By administering a single injection into the cerebrospinal fluid (CSF), di-siRNAs successfully silenced the *HTT* gene for at least 6 months in mouse models and exhibited robust silencing in both the brain and spinal cord of cynomolgus macaques [223].

However, the lack of an efficient in vivo delivery system remains a major challenge, particularly for delivering oligonucleotides to the cortex and striatum. To overcome this limitation, one study utilized focused ultrasound (FUS) in combination with an intravascular microbubble contrast agent to transiently and locally disrupt the blood–brain barrier (BBB) in the striatum of adult rats [224]. Another study loaded hydrophobically modified siRNAs (hsiRNAs) into exosomes, enabling efficient bilateral distribution when infused into the mouse striatum [225]. However, continuous delivery of siRNA into the brain and optimizing delivery efficiency are still areas for improvement.

To address these challenges, a recent study proposed a synthetic biology approach that combines the exosome-circulating system with artificial genetic circuits for the self-assembly and delivery of *mHTT*-silencing siRNA to the cortex and striatum. This strategy demonstrated an effective reduction in mHTT protein and toxic aggregates, leading to improved behavioral deficits and alleviated neuropathology in HD mouse models [226].

#### 3.3.2. MicroRNAs (miRNAs) Treatment

MiRNAs, small non-coding RNAs that regulate gene expression, have also shown promise as a therapeutic approach for HD. Studies have evaluated the efficacy and tolerability of selective or non-selective miRNA-based strategies for reducing HTT levels in mouse models. For example, intrastriatal administration of AAV5-miHTT, an AAV5 vector encoding a miRNA targeting human *HTT*, resulted in sustained suppression of HTT for at least 7 months in a mouse model [227]. Additionally, intrastriatal injection of AAV5 vectors carrying CAG repeat-targeting artificial miRNA in mice led to a significant reduction in mHTT levels and aggregates [228].

However, the translation of these findings to HD patients is hindered by differences in brain size and structure between rodents and humans. Therefore, investigating the feasibility, efficacy, and tolerability of miRNA therapy in large animal models is crucial. Studies in transgenic HD minipigs and Rhesus macaques have demonstrated the potential of miRNA therapy in reducing *mHTT* mRNA and protein levels in the brain [229,230]. Despite these promising findings, miRNA therapy still faces limitations and challenges. One major limitation is that a single miRNA often has multiple targets, which may result in unexpected side effects due to multiple bindings to different direct targets [231].

#### 3.3.3. Short Hairpin RNA (shRNA) Treatment

ShRNAs are RNA sequences that form tight hairpin structures. The transcription of the shRNA plasmid produces a product that is processed by Drosha, resulting in pre-shRNA. Exportin 5 assists in exporting this pre-shRNA from the nucleus. Dicer then processes the pre-shRNA, which binds to the RISC complex and degrades specific mRNA molecules, thereby mediating RNA interference [232]. Numerous research groups have demonstrated the effectiveness of viral vector-mediated and plasmid vector-mediated shRNAs in the long-term downregulation of HTT expression. This downregulation can be either allele-specific or non-allele-specific in various HD cell models, including patient-derived fibroblasts, iPSCs from YAC128 mice, astrocytes derived from iPSC-derived neural progenitor cells of HD monkeys, and striatal neurons derived from iPSCs of HD patients [233]. In human embryonic stem cells, Drouet et al. observed that stable knockdown of HTT also resulted in functional recovery of vesicular transport of BDNF along microtubules [234]. Franich and colleagues demonstrated that shRNAs decreased HTT expression, preventing striatal neurodegeneration and associated motor behavioral impairment in an rapid-onset HD rat model [235].

Extensive research findings suggest that suppressing both wtHTT and mHTT expression can have therapeutic benefits. However, selectively silencing *mHTT* is considered the safest approach as it preserves wtHTT expression and functions. For example, the intrastriatal injection of AAV5-mediated shRNA targeting CAG repeats resulted in a specific 50% reduction in mHTT levels and a dose-dependent decrease in HTT aggregates in the striatum of YAC128 mice [228]. In another study, selective silencing of *mHTT* was achieved using LV-mediated shRNAs targeting the single-nucleotide polymorphisms (SNPs) of the *mHTT* gene in transgenic BACHD mice and the rapid-onset HD rat model [234].

Unlike double-stranded RNA, which has a transient effect, shRNAs are delivered on a DNA plasmid, allowing for continuous expression over extended periods, ranging from months to years [232]. However, McBride et al. reported that shRNAs induce significant neurotoxicity in the striatum of HD140Q KI mice, primarily due to the robust levels of antisense RNAs arising from shRNA expression systems [236]. This finding, along with the challenge of precisely modulating shRNA expression levels, presents additional obstacles to the implementation of shRNA therapy.

#### 3.3.4. ASOs Treatment

ASOs are short nucleic acid sequences that can bind to specific mRNA sequences and recruit RNase H to degrade the targeted transcript, resulting in reduced levels of the targeted gene expression [237]. Direct administration of ASOs into the CSF through intrathecal (IT) or intracerebroventricular (ICV) injection effectively bypasses the BBB [238]. Compared to systemic administration, ICV and IT injections offer targeted drug delivery to the CNS, leading to enhanced drug availability, reduced dosage requirements, and a faster onset of action. In mice, ICV administration of Tricyclo-DNA (tcDNA) ASOs results in extensive distribution throughout the CNS. YAC128 mice, following a single ICV injection of tcDNA ASO, exhibit a widespread distribution of ASOs throughout the CNS, and this sustained reduction in mHTT levels in various brain regions lasts for up to 12 weeks [239]. To assess the efficacy of delivering ASOs into a larger, more complex brain that closely resembles the human brain, intrathecal infusion of ASOs in Rhesus monkeys is necessary. Intrathecal infusion of ASOs into the CSF of nonhuman primates leads to a sustained reduction in *HTT* mRNA in most brain and spinal cord regions, including those heavily implicated in HD pathology [240].

While ICV and IT injections target drug delivery to the CNS, it is important to note that these invasive approaches carry inherent risks, such as infection and tissue damage. Therefore, non-invasive approaches are necessary. Using apolipoprotein A-I nanodisks (apoA-I NDs) as delivery vehicles for ASOs, a single intranasal administration in BACHD mice significantly reduces mHTT levels in the cortex and striatum, the brain regions primarily affected by HD [241]. The cyclodextrin-based nanoparticles (CDs) platform is another innovative strategy to improve ASO delivery [242].

ASOs can be allele-specific, targeting *mHTT* specifically, or allele non-specific, targeting both wtHTT and mHTT. Considering that wtHTT plays a role in embryonic neurodevelopment and serves various functions in the adult brain, selectively reducing mHTT levels may be a safer option [23,243]. In the case of allele-specific ASOs, they are designed to target specific SNPs in the *mHTT* gene. Treatment with allele-specific ASOs has shown promising results in reducing cognitive and behavioral impairments in a humanized mouse model of HD, Hu97/18 [244,245].

ASOs have made significant advancements in recent years, and numerous clinical trials are currently underway to evaluate their efficacy in treating patients with HD. In a Phase III study evaluating the use of tominersen for HD treatment, there was an average reduction of 40% in specific HTT levels in the CSF of HD patients [246]. Unfortunately, due to an unsatisfactory risk/benefit assessment, the trial was halted. However, it is foreseeable that ASO-based drugs will eventually achieve optimal efficacy and minimal toxicity in the future treatment of HD.

### 3.4. Gene Editing and CRISPR/Cas System Therapy

Gene editing technology has undergone significant development in the past decade, emerging as a powerful tool for precise genome modification. These techniques allow for the targeted manipulation of specific DNA sequences, inducing double-strand breaks in the desired genomic region. Subsequently, the cell’s natural DNA repair mechanisms, such as nonhomologous end joining (NHEJ) or homology-directed repair (HDR), are activated to accurately restore the DNA. The error-prone NHEJ pathway can introduce insertion or deletion mutations in the target gene, while HDR employs an alternative DNA sequence as a template for precise repair of the break. Among the widely utilized gene editing technologies are zinc-finger nucleases (ZFNs), transcription activator-like effector nucleases (TALENs), and the CRISPR/Cas system.

Given the monogenic nature of HD, it represents an exceptional candidate for gene therapy interventions. Gene editing studies have demonstrated successful reductions in mHTT protein levels in animal models [247,248].

#### 3.4.1. ZFNs

ZFNs combine a DNA-binding domain derived from zinc fingers with a DNA-cleavage domain from the FokI endonuclease. The DNA-binding domain recognizes and binds to a specific target sequence, while the FokI domain induces a double-strand break in the DNA, enabling precise genome editing [249]. Zinc finger protein transcription factors (ZFP-TFs) offer high target specificity by designing zinc finger domains that recognize specific DNA sequences. This specificity allows for precise gene regulation. In the context of HD, ZFP-TFs have been used to selectively reduce the expression of mHTT in the brains of R6/2 HD mice and Hdh Q50 mice. This targeted reduction leads to decreased aggregation and improved motor symptoms [250,251]. However, compared to other gene editing techniques, ZFNs require more extensive design and optimization steps to achieve specific targeting and have a relatively limited target range. Therefore, addressing the limitations of these techniques in HD treatment and making efforts to overcome them is crucial.

#### 3.4.2. TALENs

TALENs utilize DNA binding domains composed of repetitive peptides that interact with DNA nucleotides, leading to the generation of double-strand breaks using artificial nucleases. This capability enables the precise deletion or correction of DNA segments. Compared to ZFNs, TALEN-based nucleases demonstrate higher efficiency and increased specificity. The implementation of an SNP-specific transcription activator-like effector (TALE-SNP) in HD patient-derived fibroblasts resulted in a significant reduction of up to 20% in *mHTT* gene expression, accompanied by a notable decrease in protein aggregation [252]. However, due to the challenges associated with delivery and the complexity involved in designing and assembling TALENs, there has been limited exploration of TALENs in HD animal models thus far.

#### 3.4.3. CRISPR/Cas9

The CRISPR/Cas system comprises the Cas9 protein and a synthetic guide RNA. The gRNA recognizes the target genomic sequence and guides the Cas9 protein to cleave the DNA double-strand, resulting in double-strand breaks that can be repaired to achieve gene knockout or gene insertion. Several research groups have successfully reduced mHTT expression in HD patient-derived fibroblasts by introducing plasmid vectors expressing CRISPR/Cas tools [253]. Han and colleagues effectively utilized CRISPR/Cas9 by designing guide RNAs to induce large deletions or frameshift indel mutations in the CAG expansion of R6/2 mouse-derived neurospheres. This approach led to a reduction in polyQ aggregation and cellular apoptosis [254]. By employing an allele non-specific strategy, researchers achieved reduced mHTT expression and aggregation, as well as improved motor symptoms in both heterozygous zQ175 mice and HD140Q-knockin mice [255,256]. Furthermore, an allele-specific strategy targeting the SNP of the *mHTT* gene resulted in a significant reduction in *mHTT* mRNA and protein levels specifically in both heterozygous zQ175 mice and BAC97 mice [257]. In 2023, Yan et al. used a Cas9-mediated strategy to replace expanded CAG repeats in the *mHTT* allele with normal CAG repeats in HD KI pigs. By introducing donor DNA containing the normal CAG repeat, they successfully depleted the *mHTT* gene, leading to significant reductions in the dysregulated expression and neurotoxicity of mHTT [258].

#### 3.4.4. CRISPR-Cas13d

CRISPR-Cas13d is an emerging type of CRISPR system specifically designed to target RNA molecules rather than DNA [259]. In comparison to shRNA, nuclear-localized sequence-fused Cas13d has shown significantly stronger RNA cleavage ability with high efficiency (approximately 96% knockdown by Cas13d compared to approximately 65% by shRNA). Additionally, Cas13d has demonstrated remarkable specificity, resulting in minimal off-target effects [260]. This innovative system holds great potential for RNA-targeted gene editing and therapeutic interventions in HD.

In a recent study, researchers developed a CRISPR-Cas13d system called Cas13d-CAGEX, which specifically targets mutant CAGEX RNA [261]. They demonstrated the effectiveness of this system by successfully eliminating toxic CAGEX RNA in fibroblasts derived from HD patients and iPSC-derived neurons. To further validate its potential, the researchers utilized an AAV vector to deliver Cas13d-CAGEX into the striatum of heterozygous zQ175 mice. This targeted delivery resulted in a selective reduction in *mHTT* mRNA and protein levels in the striatum, leading to improvements in motor coordination, attenuation of striatal atrophy, and a reduction in mHTT protein aggregates. Importantly, these positive effects were sustained for at least eight months without any adverse effects, and minimal off-target transcriptomic effects were observed. This study highlights the promising therapeutic potential of Cas13d-CAGEX in HD treatment, as it demonstrates the system’s ability to selectively target and reduce the mHTT protein, resulting in significant phenotypic improvements in an HD mouse model. However, it is crucial to note that the use of CRISPR-Cas13d in HD treatment is still in the early stages of research, and further studies are necessary to evaluate its safety, efficacy, and optimal delivery methods in clinical settings.

### 3.5. The Challenge of Gene Therapy

Numerous research findings indicate the therapeutic potential of gene editing techniques for treating HD. However, before advancing to human clinical trials, further development and comprehensive pre-clinical studies are essential. Since many current clinical trials for HD treatment use non-allele-specific gene therapy, it is important to consider the safety of this approach due to the involvement of HTT in various biological functions. While a partial reduction in normal HTT levels has been tolerated in preclinical animal models of HD, such as minipigs or adult rhesus monkeys, the long-term consequences in humans remain uncertain [262,263].

Researchers have observed that deleting the *HTT* gene causes age-dependent phenotypes. Although deletion of the *HTT* gene in mice at 2 months or earlier can cause obvious animal death because of acute pancreatitis, animal mortality is significantly reduced when the *HTT* gene is knocked out beyond four months of age [264]. Consistently, Pla et al. analyzed a mouse model in which *HTT* was selectively deleted at a young age (2 months) and found impairments in the survival and dendritic arborization of newly generated hippocampal neurons [265]. Interestingly, the absence of HTT exon 1 does not affect animal reproduction, the subcellular distribution of HTT, the expression of autophagy proteins, and global gene transcription in the mouse brain [266]. In a recent study using the KamiCas9 gene-editing system and single-nuclei RNA sequencing, researchers found that *HTT* inactivation did not induce changes in the profiles or proportions of striatal cells in aged animals, indicating a limited response to *HTT* inactivation across all cell types [267]. However, a study by Dietrich et al. found that the elimination of HTT expression in mice older than 12 months led to behavioral deficits, bilateral thalamic calcification, and disrupted brain iron homeostasis despite the absence of overt neurodegeneration [268].

Although knocking out the mouse homolog of *HTT* has been found to be embryonically lethal, inactivation of *HTT* in adult mice can significantly reduce animal mortality and does not lead to obvious alterations in neuronal survival [21,267,269,270], these results suggest that the phenotypes observed in *HTT* knockout mice are age-dependent.

In the Phase III clinical trial of tominerson, a non-allele-selective *HTT*-lowering ASO therapy, HD patients receiving tominerson experienced a more rapid worsening of composite Unified Huntington’s Disease Rating Scale scores compared to the placebo group [246]. Similarly, in another non-allele-selective *HTT*-lowering clinical trial called AMT130, three out of the 14 patients receiving the higher dose of AMT-130 experienced unexpectedly severe adverse reactions. However, at this stage, no definitive conclusions can be drawn regarding these unexpected outcomes. Researchers should consider evaluating key factors such as the potential impact of blocking the normal HTT protein on patients and the possible insufficiency of the method of administration. Based on the existing evidence, ensuring the safety of non-allele-specific therapy for HD is crucial due to the potential reduction in wtHTT levels. Identifying the most appropriate stage to intervene and the duration of treatment are critical considerations.

Significant advancements have been made in gene therapy for HD, specifically in targeting the *mHTT* alleles. This strategy aims to selectively diminish the expression of mHTT, thereby mitigating the toxic effects of the mHTT protein while maintaining the normal function of wtHTT. Such an allele-selective approach could prove particularly beneficial in treating presymptomatic young adults with HD [271].

In addition, efficient delivery of therapeutic agents across the BBB to reach affected neurons in the CNS remains a major hurdle. Researchers are actively exploring different delivery strategies and optimizing viral vectors or non-viral carriers to enhance CNS penetration. Technological advancements like microbubble-facilitated FUS combined with magnetic resonance imaging (MRI)-guided procedures have shown promise in enhancing and facilitating the targeted delivery of recombinant AAV for therapeutic gene delivery into the brain [272].

## 4. Clinical Trials for HD Treatment

Clinical trials for HD treatment are currently underway, with specific objectives aimed at evaluating the safety, effectiveness, and potential benefits of various therapeutic approaches. These approaches include RNAi, gene therapy, small molecule drugs, and non-pharmacological treatments. The ultimate goal of these trials is to improve symptoms, slow disease progression, and enhance the overall quality of life for individuals affected by HD. In the field of gene therapy for HD, several notable clinical trials and research studies have been conducted (Supplementary Materials, [273,274,275,276,277,278,279,280,281,282]).

### 4.1. Tominersen

Tominersen, developed by Ionis Pharmaceuticals, is an ASO that targets the *HTT* mRNA to reduce the production of mHTT. The GENERATION HD1 clinical trial (NCT03761849), which aimed to evaluate safety and effectiveness, has shown promising results in reducing mHTT levels in the CNS and slowing disease progression. In the Phase I/II clinical trial, tominersen significantly reduced mHTT levels, with a sustained reduction in corresponding mRNA and protein levels. After three months of treatment with the highest doses of tominersen, HD patients experienced an average reduction of 40% in specific HTT levels in their CSF. The majority of patients continued to see a decline in mHTT levels in the CSF, indicating the sustained effect of tominersen. Additionally, tominersen demonstrated good tolerability.

However, in March 2021, based on a pre-planned risk/benefit assessment conducted by an independent data monitoring committee, Roche announced the discontinuation of dosing in the Phase III study evaluating the use of tominersen for HD treatment [244]. Despite this setback, the sponsor has initiated the GENERATION HD2 trial (NCT05686551), which focuses on younger participants with a lower disease burden. In this trial, participants will receive lower doses of tominersen administered every 16 weeks without loading doses. The GENERATION HD2 trial aims to assess the potential benefits of this dosing regimen in this specific study population. The trial is expected to be completed in 2027.

### 4.2. AMT-130

UniQure, a biopharmaceutical company, is developing a gene therapy called AMT-130 that utilizes an AAV5 vector to deliver a microRNA-based therapeutic agent targeting and reducing HTT expression. Preclinical studies in animal models have demonstrated promising results in reducing mHTT levels and improving motor function [227,283]. This innovative approach of non-selective knockdown of the *HTT* gene in the brain using the AAV vector shows great potential for treating HD.

On 8 August 2022, uniQure reported that three out of 14 patients in the high-dose AMT-130 treatment group experienced severe adverse effects during a Phase 1/2 trial (NCT04120493) for HD. Following consultation with the Data Safety Monitoring Board, uniQure decided to suspend dosing in the high-dose group. Currently, a Phase 3 clinical trial called HD-KINECT (NCT04102579) is still underway to assess the safety and efficacy of AMT-130 in HD patients. In June 2023, the Phase I/II trial of AMT-130 for the treatment of HD demonstrated good tolerability and manageable safety after 24 months of follow-up. Patients showed preserved function and reductions in relevant biomarkers. Further investigation will focus on the safety of using two doses of AMT-130 in combination with perioperative immunosuppression.

### 4.3. Splice Modulators: PTC518 and Branaplam

Given the potential benefits of splice modulators in reducing the production of mHTT, several companies are actively developing splice modulators for the treatment of HD. PTC518 is an orally available small molecule specifically designed for HD. It was developed using a drug discovery platform that screened over 300,000 compounds to identify splicing modulators. PTC518 acts as a modulator of *HTT* pre-mRNA splicing by promoting the inclusion of a pseudoexon, which induces a premature termination codon and leads to the degradation of *HTT* mRNA [284,285]. This small molecule can penetrate the BBB and has shown promising results.

In June 2023, PTC Therapeutics shared mid-term data from the PIVOT-HD Phase 2 study of PTC518 (NCT05358717) in HD patients. The study demonstrated a dose-dependent reduction in HTT levels in peripheral blood cells, with an average decrease of 30% at a dose of 10 milligrams. Treatment with PTC518 was well-tolerated, with no treatment-related serious adverse events reported, and no reports of peripheral neuropathy or dose-limiting toxicity. Additionally, after 12 weeks of PTC518 treatment, the CSF NfL levels showed an overall downward trend.

Another alternative disease-modifying small molecule, branaplam, has shown promise in lowering mHTT protein levels in an HD mouse model by enhancing pseudoexon inclusion. This small molecule promotes the inclusion of non-annotated novel exons, including a frameshift-inducing exon in the *HTT* transcript. It has demonstrated a dose-dependent reduction in the total HTT and mHTT levels in patients’ fibroblasts, iPSCs, cortical progenitors, and neurons [286]. However, the occurrence of nerve damage reported in participants receiving branaplam treatment during a Phase 2 study (NCT05111249) for HD is concerning. As a precautionary measure, Novartis has decided to temporarily suspend this trial. Branaplam has been observed to alter the levels of the survival motor neuron-2 (SMN2) protein, as well as potentially other proteins, which may partially contribute to the observed side effects [287].

### 4.4. VMAT Inhibitor: SOM3355 and Valbenazine

SOM3355, also known as bevantolol hydrochloride, is a VMAT inhibitor that disrupts the transmission of dopamine messages between neurons. In a previous small trial (NCT03575676), a mixed-model analysis demonstrated a significant improvement in the total maximal chorea score, indicating its potential to effectively control HD chorea [278]. Following the completion of a clinical phase 2a trial, SOM3355 exhibited a favorable safety profile without depressive side effects. Currently, the drug is undergoing a phase 2b trial to validate its efficacy and safety in HD patients with chorea (NCT05475483).

### 4.5. Pridopidine

Pridopidine is a dopamine stabilizer that interacts with dopamine type 2 receptors. In preclinical studies, pridopidine has shown beneficial effects in R6/2 mice, including increased expression of pro-survival and neurostimulatory molecules like BDNF and DARPP32. Additionally, pridopidine treatment has led to a reduction in the size of mHTT aggregates in striatal tissues [288]. In a previous study called PRIDE-HD (NCT02006472), pridopidine demonstrated a beneficial effect on the Total Functional Capacity (TFC) for the entire population at week 52, with an improvement of 0.87 (nominal *p* = 0.0032). The effect was particularly notable in early-stage HD participants [274,275,276]. New studies suggest that pridopidine activates a protein called the sigma-1 receptor, which could have positive effects on brain health [289]. PROOF-HD (NCT04556656), a larger and longer study, aimed to examine whether pridopidine could help patients with HD maintain daily function. The study is complete, and the results are a mix of positive and negative findings. Prespecified analyses in PROOF-HD showed clinically meaningful and nominally significant benefits and improvements from baseline in disease progression, as well as motor and cognitive outcome measures when comparing pridopidine with placebo. However, these effects were not observed in patients taking neuroleptics and chorea medicines, potentially due to the masking effect of concurrent medications. As a result, the primary endpoint and key secondary endpoint did not reach statistical significance. The results from larger trials have been mixed, highlighting the complexity of HD and the need for further research to fully understand the effects of pridopidine in different patient populations and treatment contexts.

### 4.6. SAGE-718

SAGE-718, a derivative of the endogenous steroid 24(S)-hydroxycholesterol, acts as a positive allosteric modulator of the NMDA receptor. In 2021, SAGE-718 received the FDA’s fast-track designation for the treatment of HD, aiming to expedite the development and review of potentially significant new therapies. Cognitive impairment is often overlooked in HD, and currently, there are no approved treatments specifically targeting cognitive decline in affected individuals. To assess the safety and effects on cognitive function, two Phase 2 clinical trials, namely DIMENSION (NCT05107128) and SURVEYOR (NCT05358821), are evaluating SAGE-718 against a placebo in patients with HD. These trials aim to provide valuable insights into the potential of SAGE-718 as a therapeutic option for cognitive impairment in HD.

## 5. Conclusions

In recent decades, significant progress has been made in understanding the pathogenesis of HD and developing therapeutic strategies. Pharmacological interventions have shown promise in managing HD symptoms and improving patients’ quality of life. Additionally, approaches focused on modulating protein folding, enhancing protein clearance pathways, and targeting specific steps in the aggregation cascade have demonstrated encouraging results in preclinical studies. However, the translation of these promising preclinical findings into effective treatments for HD patients has proven to be challenging. Clinical trials investigating various therapeutic approaches, such as gene therapy, proteasome modulators, and antibody therapies, have encountered setbacks and have not yet produced definitive outcomes. The complexity of HD pathogenesis may be better replicated in large animals that are more similar to humans. Therefore, large animal models of HD should be considered as alternative models to validate important therapeutic targets. While gene therapy holds great promise for the effective treatment of HD, the need for precise targeting of mHTT and the difficulty of delivering therapies to the affected brain regions present significant challenges.

Despite these challenges, ongoing research and clinical trials continue to expand our understanding of HD and refine therapeutic strategies. Continuous and significant advances in understanding HD pathogenesis and developing new tools for treatment will ultimately lead to a cure for this debilitating disorder.

## Figures and Tables

**Table 1:** Pharmacogenetics of drugs used in the management of symptoms in HD.

Name of the Drug	Therapeutic Class	Mode of Action	References
Tetrabenazine	Vesicular monoamine transporter 2 (VMAT2) inhibitors	Inhibit the dopamine transporter (DAT), reduce chorea, and improve motor function	[113]
Deutetrabenazine	VMAT2 inhibitors	Inhibit the DAT, reduce chorea, improve motor function, and longer half-life	[114]
Olanzapine	Atypical antipsychotic	Management of chorea	[115]
Riluzole	Block glutamatergic neurotransmission	Improve HD associated chorea	[116]
Risperidone	Antagonistic effect of Dopamine D2 and 5HT-receptor	Control psychiatric symptoms	[117]
Amantadine	Glutamate antagonist	Reduce choreiform dyskinesias	[118]
Rivastigmine	Acetylcholinesterase inhibitor	Improve motor performances and cognition	[119]
Fluoxetine	Selective serotonin reuptake inhibitor	Management of depression and reduce agitation	[120]
Nabilone	Agonist of CB1 and CB2 cannabinoid receptors	Decrease the severity of neuropsychiatric symptoms	[121]
Benzodiazepines	Positive allosteric modulators on the GABA-A receptor	Decrease severe episodes of chorea and alleviate anxiety	[122]

## Data Availability

All data are included in the publication.

## Supplementary Materials

The supporting information can be downloaded at: https://www.mdpi.com/article/10.3390/ijms25073845/s1.
