# Supplementary material for: Huntington’s Disease: Complex Pathogenesis and Therapeutic Strategies
Source: Int J Mol Sci. 2024 Mar 29;25(7):3845. doi: 10.3390/ijms25073845 (PMC11011923; doi:10.3390/ijms25073845)
Supplement: Supplementary file 1 [file ijms-25-03845-s001.zip › ijms-2900942-supplementary.pdf]

Table S1. Notable Clinical Trials for HD Treatment

	Clinical Trials.gov ID	Trial name	Intervention	Mechanism of action	Main outcome	Phase	Sponsor	Reference
Pharmacological clinical trials	NCT02215616	LEGATO-HD	Laquinimod	Immunomodulator	Not show a significant clinical improvement	Phase 2	Teva Branded Pharmaceutical Products, R&D Inc.	[273]
	NCT02197130	Amaryllis	PF-02545920	Phosphodiesterase-10A (PDE-10) inhibitor	No improvement	Phase 2	Pfizer	-
	NCT02006472	PRIDE-HD	Pridopidine	D2R Antagonist	Improve motor function, and reduce the probability of Total Functional Capacity decline	Phase 2	Prilenia	[274-276]
	NCT04102579	KINECT-HD	Valbenazine	VMAT2 inhibitor	Improve chorea	Phase 3	Neurocrine Biosciences	[277]
	NCT03575676		SOM3355(bevantolol hydrochloride)	VMAT2 inhibitor	Reduce chorea in patients with HD, well-tolerated	Phase 2	SOM Innovation Biotech SA	[278]

NCT03225833	PRECISION-HD1	WVE-120101	Allele-selective ASO	No changes in CSF NfL	Phase 1/2	Wave Life Sciences Ltd.	-
NCT03225846	PRECISION-HD2	WVE-120102	Allele-selective ASO	Show no statistically significant change in mHTT versus placebo	Phase 1/2	Wave Life Sciences Ltd.	-
NCT05032196	SELECT-HD	WVE-003	Allele-selective ASO	Selectively led to a 22% mean reduction in mHTT while avoiding targeting the wtHTT	Phase 1b/2a	Wave Life Sciences Ltd.	-
NCT03761849	GENERATION HD1	Tominersen	ASO	Tominersen treatment shows worse scores in clinical scales compared to participants on placebo	Phase 3	Roche	-
NCT05686551	GENERATION-HD2	Tominersen	ASO	Showing dose-dependent decrease in CSF mHTT, but not benefit the participants	Phase 2	Roche	-
NCT00712426	CREST-E	Creatine	Creatine monohydrate	Not delay functional decline	Phase 1/2	Massachusetts General Hospital<br>University of Rochester<br>National Center for Complementary and Integrative Health (NCCIH)	[279]

NCT02453061	TRIHEP3	Triheptanoin	A medium-chain triglyceride indicated to provide calories and fatty acids	Correct the bioenergetic profile in the brain of HD patients	Phase 2	Institut National de la Santé Et de la Recherche Médicale, France	[280]
NCT05243017	AMT-130	AAV5-miHTT	MicroRNA-based therapeutic agent	Show positive-trending, potentially dose-dependent signals across multiple key clinical and functional measures, in conjunction with further declines in NFL	Phase 1/2	UniQure	-
NCT05111249	VIBRANT-HD	Branaplam	Splicing modulator	Show nerve damage	Phase 2	Novartis Pharmaceuticals	-
NCT04514367	ANX005	ANX-005	C1q inhibitor	Stabilize and improve functional capacity	Phase 2/3	Annexon, Inc	-
NCT02481674	SIGNAL	Pepinemab	Anti-semaphorin 4D monoclonal antibody	Reduce caudate brain atrophy and increase in FDG-PET SUVR, improve cognition in early manifest HD	Phase 2	Vaccinex Inc., Huntington Study Group	[281]
NCT05358717	PIVOT HD	PTC518	Small molecule splicing modulator	Result in a dose-dependent lowering of mHTT level	Phase 2	PTC therapeutics	-
NCT05107128	DIMENSION	SAGE-718	Positive allosteric modulator of NMDAR	-	Phase 2	Sage Therapeutics	-

	NCT04826692	-	Metformin	Antihyperglycemic/AMPK activator	-	Phase 3	Instituto de Investigacion Sanitaria La Fe	-
	NCT05541627	-	AB-1001	An adeno-associated viral vector serotype Rh10 containing the human cholesterol 24-hydroxylase gene	-	Phase 1/2	Asklepios Biopharmaceutical (AskBio) / BrainVectis	-
Non-pharmacological clinical trials	NCT04244513	-	GPi DBS	Deep brain stimulation	-	-	Beijing Municipal Administration of Hospitals, Medtronic	-
	NCT04219241	ADORE-EXT	Cellavita	Stem cell therapy	-	Phase 2/3	Azidus Brasil, Cellavita Pesquisa Cient´ifica Ltda	-
	NCT01834053	BMACHC	Bone marrow-derived MNC transplantation	Bone marrow transplant	-	Phase 1/2	Chaitanya Hospital, Pune	-

NCT03344601	PACE-HD	Physical activity	Unknown	Participants in the cohort had motor and functional decline at rates comparable to previous studies	-	Cardiff University	[282]
